# Self‐reported involvement in road traffic crashes in Kenya: A cross‐sectional survey of a nationally representative sample

**DOI:** 10.1002/hsr2.809

**Published:** 2022-09-14

**Authors:** Masood Ali Shaikh, Herman Lule, Till Bärnighausen, Michael Lowery Wilson, Anne Abio

**Affiliations:** ^1^ Injury Epidemiology and Prevention (IEP) Research Group, Department of Clinical Neurosciences, Turku Brain Injury Center Turku University Hospital and University of Turku Turku Finland; ^2^ Heidelberg Institute of Global Health (HIGH) University Hospital and University of Heidelberg Heidelberg Germany

**Keywords:** Africa, demographic health survey, epidemiology, Kenya, road traffic crashes

## Abstract

**Background:**

Road traffic crashes (RTCs) are a global public health burden whose resulting morbidity and mortality disproportionately impact low‐ and middle‐income countries with stressed health systems. There is a paucity of published studies that evaluate the sociodemographic distribution of RTCs using nationally representative samples from the African region.

**Aim:**

To examine population‐wide associations between sociodemographic factors and involvement in RTCs in Kenya.

**Methods:**

Data were obtained from the 2014 Kenyan Demographic Health Survey, representing all 47 counties in Kenya, from May to October of 2014. We estimated the prevalence of RTCs and utilized logistic regression for bivariate and multivariable analyses to determine the sociodemographic factors associated with RTCs. Study variables included age, place of residence, household wealth index, educational attainment, and history of alcohol consumption. We computed odds ratios (ORs) and their corresponding 95% confidence intervals (CIs).

**Results:**

A higher prevalence was reported among men (8.76%) versus women (3.22%). The risk factors among men included being 20−34 years of age, living in a rural area (OR 1.38, 95% CI 1.09, 1.74), drinking alcohol (OR 1.33, 95% CI 1.11, 1.59), and having not higher than a primary (OR 1.90, 95% CI 1.19, 3.03) or secondary (OR 1.68, 95% CI 1.04, 2.71) education. The strongest risk factors for women included the being aged 45−49 (OR 2.30, 95% CI 1.44, 3.67) and 20−24 years (OR 1.81, 95% 1.17, 2.79) as well as being in the fourth wealth quintile (OR 1.83, 95% CI 1.15, 2.91).

**Conclusion:**

Men and the most economically productive age groups were more likely to report being involved in RTCs. Strategies to reduce the occurrences of RTCs should prioritize the most vulnerable sociodemographic groups.

## 1 INTRODUCTION

Road traffic crashes (RTCs) are a global public health threat contributing to 9% of all causes of mortality worldwide^1^ and are a major contributor to disability‐adjusted life years lost (DALYS) from unintentional injuries. Low‐ and middle‐income countries (LMICs) disproportionately experience 85% of the mortality and 90% of DALYS associated with RTCs.^2^ The rapid motorization of transport combined with poorly planned urbanization has put many countries in the African region in a precarious situation with regard to RTCs.^3^ For instance, the African region alone is projected to record 6−14 million new cases of traumatic brain injuries resulting from RTCs in 2050.^4^ In Kenya, RTCs are the number one cause of unintentional injuries accounting for 36.8%−41.7% of all injuries and 2.4%−10% of injury‐related mortality.^5^, ^6^ In addition, injuries from RTCs in Kenya had the second highest increase in DALYS between 1990 and 2010.^7^ The lack of formalized resilient post‐injury psychosocial support systems in LMICs additionally aggravates the personal resource losses arising from RTCs beyond physical debilitation, financial incapacitation to mental distress.^8^ Saidi and Mutisto^9^ estimated the average duration of hospital stays (away from work) following RTCs in Kenya at 24.3 days at an average cost of Kenyan shillings 31,783 (US$282). Moreover, 51.7% of these patients needed surgical intervention for injuries mainly involving the limbs, head, and neck. Evidence shows that even at 3 months postsurgical intervention, most of these patients do not recover fully to their preinjury level of function.^10^

There seems to be a link between sociodemographic factors and the occurrence of RTCs, with the resulting injuries disproportionately affecting the poor.^11^ According to Odero et al.,^12^ men in the most economically productive age group (20−49) are the most affected, contributing up to 75% of injuries compared to their female counterparts. In addition, unemployed and uneducated individuals with a low‐wealth index are overrepresented among RTCs.^11^ On the other hand, road traffic injuries that affect pedestrians commonly occur in poorly designed urban areas^13^ but most fatal ones involving passengers have been reported on highways traversing rural areas, with the two vulnerable groups accounting for 80% of RTC casualties.^12^

Knowledge of the most vulnerable populations is key to targeted preventive policy, particularly in LMICs with constrained budgets dedicated to the health sector. Kenya is one of such countries transitioning from an infectious, maternal, and child health disease burden to a noncommunicable and injury‐related morbidity and mortality burden. According to Bachani et al.,^14^ the country experienced a road traffic injury rate of 60 per 100,000 population as of 2009, mainly resulting from speeding motor vehicles and motorcycles. Moreover, injury statistics are often underreported in Kenya due to a lack of robust trauma data registries.^15^ The lack of preventive education campaigns, poorly monitored and uncoordinated road safety interventions, lack of coalition among road safety stakeholders, an increasing number of motorcycles, and poorly designed roads in congested urban areas ‐ are major contributing factors to RTCs in Kenyan settings.^9^, ^12^, ^13^ For instance, whereas 57%−58% of cars in Kenya's main cities are driven above local speed limits,^7^ seat belt use among drivers and passengers is reported at 12.5%.^1^ Furthermore, helmet use is at 33% and 3% among motorcyclists and their passengers, respectively,^14^ despite the known severe head injury risk for non‐helmet use.^16^ In addition, only 8.5% of road traffic crash incidents are reported to police for legal action^17^ with 8.1% of RTCs in Kenya being at least partly linked to alcohol‐influenced driving.^1^ Unfortunately, the legal enforcement of the traffic act in Kenya has not yielded any reduction in injury severity.^18^

Despite the poor trauma care indices in the country,^19^ Kenya is regarded as one of the countries in the African region with an improved prehospital care system,^20^ signifying the disproportionate unmet need for trauma care in the region. However, there is a paucity of published studies that have evaluated the sociodemographic distribution of RTCs in Kenya which involve nationally representative samples. A similar study by Odhiambo et al.,^19^ was limited to general trauma‐related mortality in rural western Kenya. The present study explored RTCs and associated sociodemographic variables using a nationally representative sample from the Kenyan contribution to the Demographic and Health Survey (2014).

## 2 METHODS

### 2.1 Study design

We used previously collected Kenyan Demographic and Health Survey 2014 (KDHS‐2014) data to inform this study. DHS surveys have been conducted in 91 LMICs in collaboration with each country's national agencies and the DHS program. DHS surveys are one of the only or the most recent nationally representative surveys available on health and demographic indices in participating countries. The KDHS‐2014, for the very first time, provided national estimates on RTC events as well as on other unintentional injuries. The KDHS‐2014 is the sixth DHS conducted in the Republic of Kenya. Additional information is available elsewhere.^21^

### 2.2 Sampling approach

A two‐stage cluster sampling design was used in KDHS‐2014 for providing a representative sample on a range of health and population indices, including involvement in RTCs at the national level, for urban and rural areas separately, and at the regional/provincial levels. Data were collected from all 47 counties from clusters sampled with a probability proportional to the size sampling methodology. From a total of 96,251 enumeration areas, based on the 2009 Kenya Population and Housing Census, in the first stage, 1,612 clusters were selected (995 rural and 617 urban areas), while in the second stage, 25 households were randomly selected from each cluster, resulting in a total of 39,679 households selected for the final sample.^22^

### 2.3 Data collection

The survey was conducted by the Kenya National Bureau of Statistics in partnership with the Ministry of Health. The data collection phase of the KDHS‐2014 was done from May to October 2014.^21^ KDHS‐2014 asked the question, “In the past 12 months, have you been involved in a road traffic accident as a driver, passenger, pedestrian, or a cyclist?”. The responses were coded as either “yes” or “no.” Cumulatively, 14,730 women aged 15−49 years and 12,816 men aged 15−54 years answered the question on RTC involvement in the 12 months preceding the survey.

### 2.4 Study variables

A single question in the KDHS‐2014 inquired about involvement in RTCs. This outcome variable was determined from the question, “In the past 12 months, have you been involved in a road traffic accident as a driver, passenger, pedestrian, or a cyclist?”. Affirmative answers were coded as 1 and negative answers were coded as 0.

Five explanatory variables of age, place of residence (urban/rural residency status), household wealth index, educational attainment, and alcohol use were used to study the associations with involvement in RTCs.

#### 2.4.1 Age

Age was grouped into 5‐year intervals from 15−19, 20−24, 25−29, 30−34, 35−39, 40−44, 45−49, and 50−54 years. The lowest age group of 15−19 years was used as the reference group in the bivariate and multivariable analyses.

#### 2.4.2 Residence

Residency status in terms of urban or rural was examined. The urban residency status was used as the reference group in the bivariate and multivariable analyses.

#### 2.4.3 Wealth index

Each household's wealth index was calculated in the KDHS‐2014 based on ownership of certain assets and categorized into five groups, that is, quintiles ranging from “lowest,” “second,” “middle,” “fourth,”, and “highest.” The lowest age wealth quintile was used as the reference group in the bivariate and multivariable analyses.

#### 2.4.4 Educational attainment

Four categories were used to determine educational attainment ranging from “no education,” “primary education,” “secondary education,” and the final category entailing “higher” educational attainment. The no education group was used as the reference group in the bivariate and multivariable analyses.

#### 2.4.5 Alcohol use

This was determined from the KDHS‐2014 question, “Do you drink alcohol?”, with binary response options of either “yes” or “no.” The alcohol nonuser group was used as the reference group in the bivariate and multivariable analysis.

### 2.5 Data analysis

The “women” and “men” data sets of the KDHS‐2014 were downloaded in the STATA format from the DHS program website, after obtaining written permission.^21^ The complex survey design of the KDHS‐2014 was taken into account. Proportions, presented as percentages, were calculated separately for both sexes, including their 95% confidence intervals (CIs). Associations between the RTCs and sociodemographic characteristics, that is: age, place of residence in terms of urban/rural residency status, household wealth index, and educational attainment, were determined using simple binary logistic regression models. Additionally, the association between alcohol use and RTCs was also determined. Results are reported as odds ratios and their corresponding 95% CIs. Variable associations that were found to be statistically significant at the level of *p* < 0.05 were included in the final multivariable logistic regression model, and adjusted odds ratios and their 95% CIs are reported. The final multivariable models were found to have a good fit based on the Hosmer–Lemeshow test for survey data.^23^ All analysis was conducted using STATA version 16 (StataCorp).

### 2.6 Ethical considerations

KDHS‐2014 was granted ethical approved by the Kenya Medical Research Institute. Data were collected after receiving informed consent from every participant.^22^ For this study, ethical approval was not applicable since this is a secondary analysis of the publicly available data.

## 3 RESULTS

A total of 440 women (3.22%: 95% CI 2.80−3.69) and 1123 men (8.76%: 95% CI 8.02−9.57) reported having sustained an RTC in the 12 months preceding the survey.

**Figure 1:**
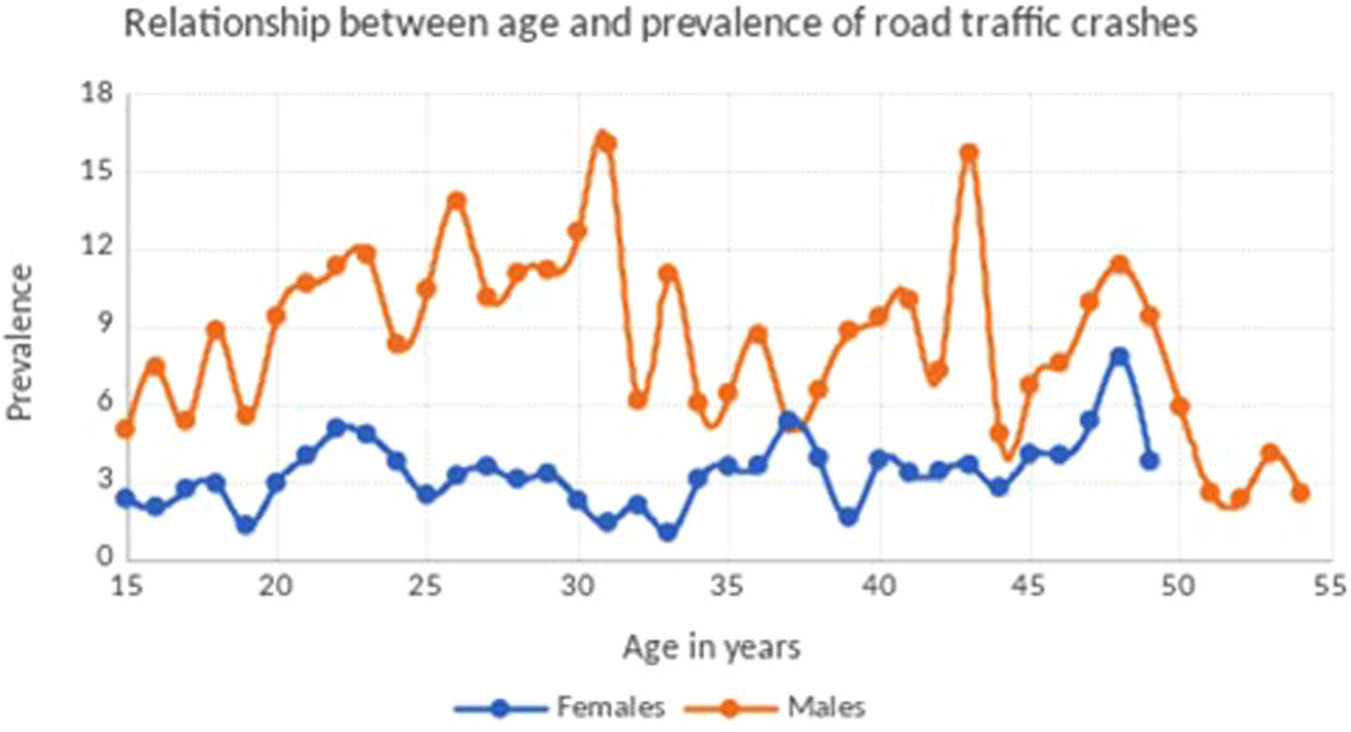
Relationship between age and prevalence of road traffic crashes in Kenya (2014).

Table 1 shows the results of the exploratory data analysis in terms of RTC proportions and their corresponding 95% CIs, for both women and men disaggregated by the five explanatory variables, and their unweighted counts. The highest prevalence of RTCs in women was found in the 45–49‐year age group (4.93%), while the lowest prevalence was in the 30–34‐year age group (2.05%). In men, the highest prevalence was reported by the 25–29‐year‐old group (11.29%), while the lowest prevalence was reported by the 50–54‐year‐old group (3.67%). The relationship between age and the occurrence of RTCs is presented in Figure 1. Regarding residency status, women living in urban areas reported a higher RTC prevalence (3.72%), compared to their rural counterparts. On the other hand, men living in rural areas reported a higher RTC prevalence (9.59%), compared to men from urban areas.

**Table 1:** Prevalence, 95% confidence intervals, and unweighted counts of road traffic crashes in Kenya disaggregated by explanatory variables (Kenya DHS‐2014)

	Women (*N* = 14,730)				Men (*N* = 12, 816)			
Variable	Prevalence %	95% CI	rRTC	nRTC	Prevalence %	95% CI	rRTC	nRTC
Age								
15−19	2.27	1.70−3.03	67	2791	6.51	5.50−7.70	183	2627
20−24	4.13	3.02−5.63	83	2453	10.18	8.72−11.85	220	1761
25−29	3.14	2.22−4.42	82	2775	11.29	9.55−13.30	224	1718
30−34	2.05	1.42−2.95	51	2056	10.40	8.43−12.78	165	1535
35−39	3.65	2.65−5.01	60	1814	7.20	5.79−8.91	126	1360
40−44	3.44	2.53−4.65	54	1316	9.27	7.03−12.13	104	1093
45−49	4.93	3.37−7.16	43	1085	8.74	6.31−11.98	70	825
50−54	N/A				3.67	2.39−5.58	31	774
Residence								
Urban	3.72	2.92−4.73	189	5277	7.68	6.35−9.25	418	4496
Rural	2.87	2.48−3.33	251	9013	9.59	8.83−10.42	705	7197
Education								
No education	1.34	0.65−2.71	26	1952	5.39	3.50−8.19	29	737
Primary	3.16	2.66−3.74	223	7178	9.64	8.68−10.69	640	5909
Secondary	3.54	2.75−4.53	148	3953	8.25	7.07−9.59	334	3726
Higher	3.76	2.53−5.54	43	1207	7.76	6.00−9.98	120	1321
Wealth quintile								
Lowest	1.89	1.41−2.54	67	3331	8.71	7.32−10.33	197	2484
Second	3.19	2.44−4.16	86	2778	9.79	8.40−11.37	241	2337
Middle	3.11	2.43−3.98	88	2753	10.03	8.60−11.66	269	2367
Fourth	4.17	3.16−5.49	105	2734	8.63	7.20−10.30	236	2521
Highest	3.31	2.36−4.64	94	2694	7.12	5.56−9.08	180	1984
Alcohol use								
No	3.11	2.71−3.58	411	13,717	8.00	7.22−8.87	745	8628
Yes	5.28	3.21−8.56	29	573	10.52	9.21−12.00	378	3059^a^
Total	3.22	2.80−3.69	440	14,290	8.76	8.02−9.57	1123	11,693

Abbreviations: CI, confidence interval; nRTC, nonreported RTCs; rRTC, reported RTCs.

a Six records with missing data.

For educational attainment, women with higher educational status reported the highest RTC prevalence (3.76%), and those with no education reported the lowest (1.34%). For men, the highest prevalence was reported by those with primary education (9.64%), and those with no education the lowest (5.39%). For household wealth index in terms of quintile, the highest RTC prevalence was reported by women in the fourth quintile (4.17%), and the lowest prevalence was reported by those in the lowest wealth quintile (1.89%). In men, the highest RTC prevalence was reported in the middle quintile (10.03%), and the lowest in the highest wealth quintile. For both women as well as men, those who reported use of alcohol had a higher RTC prevalence with 5.28% in women and 10.52% in men.

Tables 2 and 3 show the results of simple and multivariable logistic regression models in terms of crude odds ratios (OR), adjusted odds ratios (aOR), their statistical significance, and the associated 95% CIs for women and men, respectively. Based on the results of the binary simple logistic regression models: out of five explanatory variables, among women residency status in terms of urban/rural residency was not found to be statistically significant, while in men household wealth index was not statistically significant. For each gender, a separate multivariable binary logistic model only included those explanatory variables that were found to be statistically significant.

**Table 2:** Crude and adjusted odds ratios for the association between road traffic crashes with explanatory variables in women in Kenya (DHS‐2014)

Variable	cOR	95% CI	*p* value	aOR	95% CI	*p* value
Age						
15−19	Ref.			Ref.		
20−24	1.85	1.90−2.88	0.006	1.81	1.17−2.79	0.008
25−29	1.39	0.88−2.21	0.159	1.38	0.87−2.18	0.175
30−34	0.90	0.56−1.45	0.659	0.89	0.53−1.48	0.658
35−39	1.63	1.04−2.56	0.034	1.66	1.04−2.66	0.034
40−44	1.53	0.99−2.36	0.055	1.53	0.98−2.39	0.062
45−49	2.23	1.42−3.50	0.001	2.30	1.44−3.67	0.001
Residence						
Urban	Ref.			N/A		
Rural	0.77	0.57−1.02	0.072			
Education						
No education	Ref.			Ref.		
Primary	2.41	1.14−5.10	0.021	2.14	0.96−4.81	0.065
Secondary	2.71	1.26−5.84	0.011	2.32	0.98−5.47	0.056
Higher	2.88	1.26−6.61	0.012	2.29	0.93−5.63	0.072
Wealth quintile						
Lowest	Ref.			Ref.		
Second	1.71	1.14−2.57	0.010	1.45	0.94−2.26	0.095
Middle	1.67	1.13−2.46	0.011	1.41	0.89−2.21	0.141
Fourth	2.25	1.48−3.43	*<*0.001	1.83	1.15−2.91	0.010
Highest	1.78	1.12−2.82	0.015	1.39	0.83−2.32	0.207
Alcohol use						
No	Ref.			Ref.		
Yes	1.73	1.02−2.94	0.041	1.67	0.98−2.84	0.059

Abbreviations: aOR, adjusted odds ratio; CI, confidence interval; cOR, crude odds ratio; DHS, Demographic and Health Survey; Ref., reference.

**Table 3:** Crude and adjusted odds ratios for the association between road traffic crashes with explanatory variables in men in Kenya (DHS‐2014)

Variable	cOR	95% CI	*p* value	aOR	95% CI	*p* value
Age						
15−19	Ref.			Ref.		
20−24	1.63	1.28−2.07	*<*0.001	1.72	1.34−2.20	*<*0.001
25−29	1.83	1.41−2.36	*<*0.001	1.89	1.45−2.46	*<*0.001
30−34	1.67	1.24−2.25	0.001	1.65	1.22−2.23	0.001
35−39	1.11	0.83−1.50	0.479	1.10	0.81−1.49	0.544
40−44	1.47	1.04−2.06	0.027	1.44	1.01−2.04	0.042
45−49	1.37	0.93−2.02	0.107	1.32	0.88−1.97	0.183
50−54	0.55	0.34−0.88	0.012	0.52	0.32−0.83	0.007
Residence						
Urban	Ref.			Ref.		
Rural	1.28	1.02−1.60	0.032	1.38	1.09−1.74	0.008
Education						
No education	Ref.			Ref.		
Primary	1.87	1.17−2.99	0.009	1.90	1.19−3.03	0.008
Secondary	1.58	0.98−2.56	0.063	1.68	1.04−2.71	0.036
Higher and above	1.48	0.87−2.52	0.150	1.44	0.84−2.48	0.184
Wealth quintile						
Lowest	Ref.			N/A		
Second	1.14	0.88−1.47	0.318			
Middle	1.17	0.91−1.50	0.220			
Fourth	0.99	0.75−1.31	0.943			
Highest	0.80	0.58−1.12	0.191			
Alcohol use						
No	Ref.			Ref.		
Yes	1.35	1.14−1.60	*<*0.001	1.33	1.11−1.59	0.002

Abbreviations: aOR, adjusted odds ratio; CI, confidence interval; cOR, crude odds ratio; DHS, Demographic and Health Survey; Ref., reference.

As shown in Table 2, in the multivariable model for women, out of four explanatory variables, only age and wealth index were found to be statistically significantly associated with RTCs. Within the age variable, the age groups 20−24 (aOR, 1.81: 95% CI 1.17−2.79, *p* value 0.008), 35−39, (aOR, 1.66: 95% CI 1.04−2.66, *p* value 0.034) and 45−49 (aOR, 2.30: 95% CI 1.44−3.67, *p* value 0.001) were statistically significantly associated with RTCs, compared to the age group 15−19 years. For wealth index variable, only the fourth quintile (aOR, 1.83: 95% CI 1.15−2.91, *p* value 0.010) was statistically significantly associated with RTCs.

As shown in Table 3, in the multivariable model for men, four explanatory variables were found to be statistically significantly associated with RTCs. Within the age variable, the age groups of 35−39, 40−44 and 45−49 years were not found to be statistically significantly associated with RTCs, compared to the age group 15−19 years. The age groups of 20−24, 25−29, and 30−34 years experienced an increased odds of having an RTC compared to the 15–19‐year age group. However, for the age group 50−54 years, the odds of having an RTC decreased by half (aOR, 0.52: 95% CI 0.32−0.83, *p* value 0.007) compared with the 15–19‐year age group. For the educational attainment category, only the higher educational attainment group was not statistically significantly associated with RTCs, compared with the group with no educational attainment.

## 4 DISCUSSION

The study aimed to estimate the prevalence of RTCs and the associated factors. The prevalence was 3.22% among women and 8.76% among men. A higher prevalence was also observed among the economically productive age groups in both women and men.

We found that age groups lying within (20−24, 35−39, and 45−49) years for women and (20−34) years for men were associated with RTCs compared to the age group (15−19) years. Other studies have reported higher RTC incidence, especially among men in the age group (20−49) years.^9^, ^14^, ^17^, ^24^, ^25^ Studies from the region (Kenya and Seychelles) have shown that men are four times more likely to die from RTC‐related trauma.^19^, ^26^ The combination of being male and of working age, in the African context, means that families are often deprived of household breadwinners.^27^ With respect to age, whereas the older age groups are less likely to get involved in RTCs, in case they do, from the Kenyan experience, they are more likely to die within the hospital.^5^, ^19^ From a low‐resourced setting perspective, such mortality could result from the frailty of the elderly population, underlying comorbidities, injury severity, or rather simply from inadequate post‐crash care. In terms of dealing with the aftermath of potential injuries arising from RTCs, Kenya experiences shortages of basic medical supplies for trauma care with only 40.8% of its health facilities being adequately prepared to handle road‐traffic injured patients.^25^ In contrast to its rural areas, health facilities within Kenya's capital city of Nairobi are reported to have adequate trauma care infrastructure in terms of physical and human resources, with 96% of facilities in the capital meeting the WHO minimal safety criteria.^28^

However, it should be pointed out that while 19% of injuries resulting from RTCs in Kenya are severe,^29^ only 1.4% of RTC casualties are transported by ambulance and only 49.6% of such patients arrive at trauma centers within 1 hour following the trauma incident.^15^ In addition, only 7.4%−16% are likely to have received any form of first aid as the majority of patients are transported to hospitals by unidentified lay individuals.^15^, ^25^ This paints a clearer picture as to why the country has a high proportion of prehospital deaths from injuries at 51.4%, while simultaneously having a much lower percentage of in‐hospital deaths being due to injuries at 4.4%. Autopsies are performed in only 19% of trauma deaths to ascertain the cause.^6^, ^15^

Men residing in rural areas were more likely to report RTCs. However, a higher risk among urban dwellers was reported in a nationally representative Ethiopian study.^30^ As opposed to our study that only had information on RTC events with no mention of injuries, the Ethiopian study collected information about road traffic casualties, for instance, resulting in death or severe injuries.^30^ It is possible that a number of the RTC events in Kenya happened along rural roads due to inadequate adherence to traffic laws. Traffic laws are less likely to be enforced in rural areas where there is a limited presence of traffic police.^11^, ^31^ Approximately 60% of RTCs have occurred on rural roads in Kenya.^12^ On the other hand, it was not possible to determine the location where the RTC events occurred in this study nor examine subpopulations such as pedestrians, passengers, or drivers.

For the wealth index variable, women in the fourth quintile were 1.8 times more likely to be involved in an RTC event. An Ethiopian study using the 2016 DHS data also found a higher risk among the higher wealth quintiles.^30^ Globally, the occurrence of unintentional injuries tends to decline with increasing social demographic index^32^; however, the road traffic injuries seem to follow an opposite trend.^1^ This might be attributable to automobile ownership which is reported to be high among individuals with a higher wealth index,^33^ but because the casualties might be pedestrians, passengers, or drivers, this association might be hard to ascertain.

In addition, we found that RTC events were significantly associated with men with primary and secondary educational attainment but not women. In a similar study by Bachani et al.,^34^ education and being in school were protective against injuries even after controlling for age, gender, occupation, and the country's income profile. However, in this Kenyan study, the increased risk among the men with primary and secondary education could be attributed to insufficient exposure to safe traffic practices. Having minimal educational attainment as a risk to RTCs might be due to inadequate exposure to preventive education information such as road traffic signs that are largely embedded into the country's standard school curriculum. Individuals with inadequate exposure to road traffic safety information are 1.3 times more likely to be involved in RTCs.^17^ In addition, the psychosocial stress and substance abuse among the uneducated poor could contribute to their vulnerability to RTCs.^35^ African men are twice as likely to engage in substance use compared to their female counterparts^36^ and for Kenya's situation, this assertion is true even at the time of the road traffic crash.^37^

Men who consumed alcohol were more likely to report RTCs. Gathecha et al.,^6^ found no association between alcohol consumption and road traffic injuries in a nationally representative sample in Kenya. In this study, it was not possible to ascertain whether the consumption of alcohol had a direct influence on the RTCs reported. The Kenyan national alcohol consumption rate is reported at 13.3%^38^ and there are instances of drunk driving according to the police reports.^11^ Drunk driving, in combination with other environmental and behavioral factors or vehicle characteristics, is likely to exacerbate RTC incidents.^11^ Behavioral changes to improve road safety in general are still required to reduce the incidence of RTCs and safeguard the population to minimize avoidable injuries which may arise.

### 4.1 Study limitations

The present study was not without limitations. First, the data are silent on the involvement of children and adolescents less than 15 years of age in traffic collisions. RTCs are known to be one of the most significant contributors to traumatic brain injuries among children outside of the home environment.^34^, ^39^ Second, information concerning the location of the RTC event was not captured in the survey, which would be a useful consideration when designing targeted strategies for prevention efforts. Third, the question about the RTCs asked in the questionnaire was not sufficiently explicit in its ability to capture injuries as a result of the RTC as opposed to only RTC event information. RTC events in and of themselves are not equivalent to injuries sustained in the road traffic environment. For this study, it was not possible to estimate the prevalence of injuries as a result of the RTCs. Fourth, the questionnaire did not assess the use of illicit substances. The use of illicit substances could potentially influence RTCs and it may be worthwhile to collect information about their use in future surveys. Finally, assessing alcohol intake via questionnaires has been shown to be imprecise,^40^ and it is this imprecision that has a tendency to drive associations toward the null.

## 5 CONCLUSION

The present study has revealed that men and the economically productive age groups were more likely to report RTCs. Higher odds of RTCs were also reported by women in the fourth wealth quintile and men with primary or secondary levels of education as well as those who consumed alcohol. There is an urgent need to emphasize injury control measures that simultaneously take into consideration the socio‐economic gradients that underpin and influence human behavior. These aspects are important drivers of RTCs at the population level.

## AUTHOR CONTRIBUTIONS

*Conceptualization*: Anne Abio. *Formal analysis*: Anne Abio, Masood Ali Shaikh. *Methodology*: Anne Abio, Masood Ali Shaikh. *Validation*: Anne Abio, Masood Ali Shaikh, Herman Lule, Till Bärnighausen, Michael Lowery Wilson. *Writing original draft*: Masood Ali Shaikh, Herman Lule, Till Bärnighausen, Michael Lowery Wilson. *Writing—review and editing*: Anne Abio, Herman Lule, Till Bärnighausen, Michael Lowery Wilson, Masood Ali Shaikh. *Supervision*: Michael Lowery Wilson. All authors have read and approved the final version of the manuscript.

## CONFLICT OF INTEREST

The authors declare no conflict of interest.

## TRANSPARENCY STATEMENT

The lead author had full access to all of the data in this study and takes complete responsibility for the integrity of the data and the accuracy of the data analysis.

## Data Availability

The data that support the findings of this study are available on the Demographic Health Survey (DHS) website at https://dhsprogram.com. These data can be accessed after a request is submitted and permission to use the data is granted by the DHS team.
